# Clinicopathological and molecular features of gynecologic perivascular epithelioid cell tumors: a single-center study

**DOI:** 10.3389/fonc.2026.1769702

**Published:** 2026-02-18

**Authors:** Heng Li, Xiaoteng Sun, Zhihui Hou, Rui Xin, Chunrong Li, Li Li

**Affiliations:** 1 Department of Pathology, School of Basic Medical Sciences and Qilu Hospital, Shandong University, Jinan, Shandong, China; 2 Department of Pathology, Rushan People’s Hospital, Rushan, Shandong, China; 3 Department of Pathology, Qilu Hospital (Qingdao), Shandong University, Qingdao, Shandong, China; 4 Department of Pathology, Hospital for Reproductive Medicine Affiliated to Shandong University, Jinan, Shandong, China; 5 Department of Pathology, Qilu Hospital, Shandong University, Jinan, Shandong, China

**Keywords:** gynecologic, immunohistochemistry, PEComa, TFE3, TSC2

## Abstract

**Background:**

Perivascular epithelioid cell tumors (PEComas) are rare neoplasms characterized by the expression of both melanocytic and myoid markers. Gynecologic PEComa may show overlapping with smooth muscle tumors and other uterine tumors. The rarity and ill-defined risk stratification make the diagnosis challenging. The current study was aimed to more fully characterize gynecologic PEComas.

**Methods:**

We investigated the clinicopathological and immunohistochemical features of nine gynecological PEComas from a single center. The molecular landscape was assessed using a combined DNA-RNA hybrid capture-based comprehensive genomic profiling assay.

**Results:**

Patients, aged from 27 to 79 years old, presented with the disease predominantly located in the uteri (5/9) and cervix (3/9). Histologically, six cases were classified as malignant and three as having uncertain malignant potential. All tumors were composed of epithelioid or spindled cells arranged around the vessels, with variable nuclear atypia and necrosis. Immunohistochemical analysis revealed universal HMB45 and TFE3 positivity, and variable myoid marker expression. HMB45 was confirmed as the most sensitive diagnostic marker. Mutually exclusive genetic alterations were identified in all six tested cases with TSC2 mutations/deletions in three tumors and TFE3 fusions in another three. And here we reported a novel YAP1-TFE3 gene fusion in gynecological site. It was found TSC2-altered tumors harbored more additional mutations, whereas TFE3-rearranged tumors occurred in younger patients. Most cases showed malignant potential or malignancy, necessitating risk stratification according to the current WHO algorithms.

**Conclusions:**

This study highlights the key histological and molecular features, and advocates for integrated DNA/RNA sequencing to guide prognosis and targeted therapies.

## 1 Introduction

Perivascular epithelioid cell tumors (PEComa) is a rare mesenchymal tumor composed of perivascular epithelioid cells (PEC) and belongs to a family that includes angiomyolipoma, clear cell “sugar” tumor of the lung, lymphangiomyomatosis, and a group of uncommon lesions with similar morphology and immunophenotype occurring in soft tissues and visceral organs (1–3). PEComas coexpress melanocytic and smooth muscle markers, and are often closely associated with blood vessel walls, although their origin remains unclear (3–6). Gynecologic PEComa was first reported by Pea et al. in 1996 (4), and then was recognized by the World Health Organization (WHO) classification of gynecologic tumors in 2003 (7). To date only approximately 150 cases have been reported to date (8–11). Owing to its rarity and overlapping morphology and immunophenotype, PEComa is often confused with epithelioid smooth muscle tumors (ESMT), endometrial stromal sarcoma(ESS), malignant melanoma, rhabdomyosarcoma, inflammatory myofibroblastic tumor(IMT), and even undifferentiated carcinoma, making the diagnosis challenging. Furthermore, algorithm for prognostication and a protocol for the treatment of this neoplasm have yet to be firmly established. More extensive study and exploration of their clinicopathological features and molecular genetic alterations are of great significance for improving our understanding of this tumor in female genital tract.

In this study, the morphological and immunohistochemical features of nine gynecologic PEComas with follow-up data from a single center were evaluated. For the first time, a comprehensive genomic profiling using both DNA-based and RNA-based next generation sequencing (NGS) was performed to detect the genetic alterations in gynecologic PEComa. In addition, YAP1-TFE3 gene fusion was observed in gynecologic PEComa firstly.

## 2 Materials and methods

### 2.1 Case selection

In this retrospective study, 9 gynecologic PEComas from January 2017 to December 2022 were collected by searching the pathology system from the institutional and consultation archives of the Department of Pathology of Qilu Hospital. None of the identified cases in the searching were excluded. Gross findings were recorded according to the pathology reports. There were 6 institutional cases and 3 consultation cases. All surgical resection specimens were fixed in 4% neutral formaldehyde, routinely embedded in paraffin, sectioned, and stained with hematoxylin and eosin (H&E) for light microscopic observation. 15 serial sections (4 µm) were prepared for each tumor and used for further immunohistochemical staining. When available, another 10 sections of 5μm were used for targeted sequencing. The use of archived diagnostic tissues for research purposes as well as patient data analysis has been approved by the Institutional Review Board (ECSBMSSDU2019-1-25). All studies were conducted in compliance with the Helsinki Declaration.

### 2.2 Pathological examination

For each case, the original H&E and immunohistochemically stained slides were reviewed by two experienced pathologists (L.L. and XT.S.) to confirm the diagnosis based on the 5th WHO established definition of PEComa (12). Histological grading was also performed according to the WHO algorithm. Tumors were diagnosed as malignant PEComa if they exhibited at least three of the following features: size >5 cm, significant atypia, mitotic figure >1/50 per high power field (HPF), necrosis and lymphovascular space invasion. Cases with fewer than three of the above features were recognized as uncertain malignant potential (UMP) (12). Nuclear atypia was graded as mild or significant based on the uniformity of cell size and shape, regularity of cell shape, coarseness of chromatin, thickness of the nuclear membrane, and size of the nucleoli.

### 2.3 Immunohistochemistry

Immunohistochemical staining was performed on serial paraffin sections. The sections were deparaffinized with xylene and rehydrated through a graded alcohol series. After rinsing in 10mM TRIS-EDTA buffer in the microwave oven for antigen retrieval, sections were treated with 3% hydrogen peroxide in methanol to eliminate endogenous peroxidase activity. Then primary monoclonal antibody (ready-to-use) against HMB45(clone HMB45), MelanA (OTI12H10), SMA (UMAB237), Caldesmon (EP19), desmin (OTIR4A8), TFE3(EP285), S-100(15E2E2 + 4C4.9), CKpan(AE1/AE3), EMA (GP1.4), Vimentin (UMAB159) and ki-67 (UMAB107) from Zhongshan Bio-Tech(Beijing), ER (SP1, Roche) and PR(1E2, Roche) were applied to the sections. After washing, the primary antibody was detected with appropriate secondary antibody for 30 min at 37 °C.Detection was performed using avidin–biotincomplex and visualized by 3, 3′-diaminobenzidine (DAB). The intensity of staining was interpreted as weak (1+), moderate (2+) and strong (3+). The extent of staining was categorized as <1% expression (negative), 1% to 10% (very focal), 11% to 50% (focal), and > 50% (diffuse). Positive sections produced in preliminary experiments were used as a positive control. Phosphate Buffered Saline (PBS) was used instead of the antibody as a negative control.

### 2.4 NGS

We profiled 6 PEComas for genomic alterations using both DNA-based and RNA-based NGS. The specimens of the other three consultation cases were not accessible, so NGS testing was not conducted. Targeted-capture NGS was performed with DNA using a 481-gene panel for soft tissue (SARCOPACT™, Geneseeq Technology Inc., Nanjing) to detect single nucleotide variants (SNVs), small insertions and deletions (indels), copy number variations (CNVs), and selected gene fusions, tumor mutation burden (TMB), microsatellite instability (MSI).Targeted RNA sequencing was performed using a 149-gene panel for soft tissues (Sarcorna™, Geneseeq Technology Inc., Nanjing) to detect significant gene fusions. The details were shown in **Supplementary Materials** and Methods.

### 2.5 Follow-up

Follow-up data, including tumor recurrence, metastasis, survival, and current treatment, were obtained by telephone interviews or institutional medical records. The follow-up period was defined as the period from hospital discharge to the date of patient’s death or the last follow-up.

## 3 Results

### 3.1 Clinical features

The average age of the nine patients was 51.2 years (median: 51 years), ranging from 27 to 79 years old. Five patients underwent hysterectomy and bilateral salpingo-oophorectomy, three underwent tumor resection, and one had debulking surgery due to the invasion of the iliac vessels. Adjuvant chemotherapy or radiotherapy was administered to 3 patients. 5 tumors were located in the uterine corpus, 3 in the cervix, and 1 from in the vagina. According to the aforementioned criteria, 6 out of 9 tumors were diagnosed as malignant PEComa, while the remaining 3 were classified as UMP. Notably, one of these UMP cases would have been categorized as benign PEComa according to the 2014 WHO criteria (13) (see **Table 1**).

**Table 1:** Clinical features of 9 gynecologic PEComas.

Case#	Age	Location and extent	No. of nodules	Size(cm)	Treatment	Follow-up duration(m)	Outcome
1	56	Uterus corpus with direct extension to iliac vessels	Multiple	7.8	Suboptimal debulking surgery	43	DOD
2	27	Vagina with involvement of bladder	Multiple	5.8	Excision; adjuvant chemotherapy	62	Alive, LR
3	36	Cervix	Single	4.4	H-BSO; adjuvant chemotherapy	38	Alive, NED
4	79	Uterus corpus	Single	7.2	H-BSO	35	DOD
5	57	Cervix	Single	5.5	H-BSO; adjuvant radiotherapy	41	Alive, LR
6	35	Uterus corpus	Single	16.6	Excision	36	Alive, NED
7	47	Cervix extending to vagina	Single	3.8	H-BSO + vaginectomy	40	Alive, NED
8	73	Uterus corpus	Single	4.5	H-BSO	35	Alive, NED
9	51	Uterus corpus	Single	5.5	H-BSO	40	Alive, NED

M, malignant; UMP, uncertain malignant potential; DOD, died of disease; H-BSO, hysterectomy and bilateral salpingo-oophorectomy; LR, local recurrence; NED, no evidence of disease.

### 3.2 Pathological features

**Figure 1:**
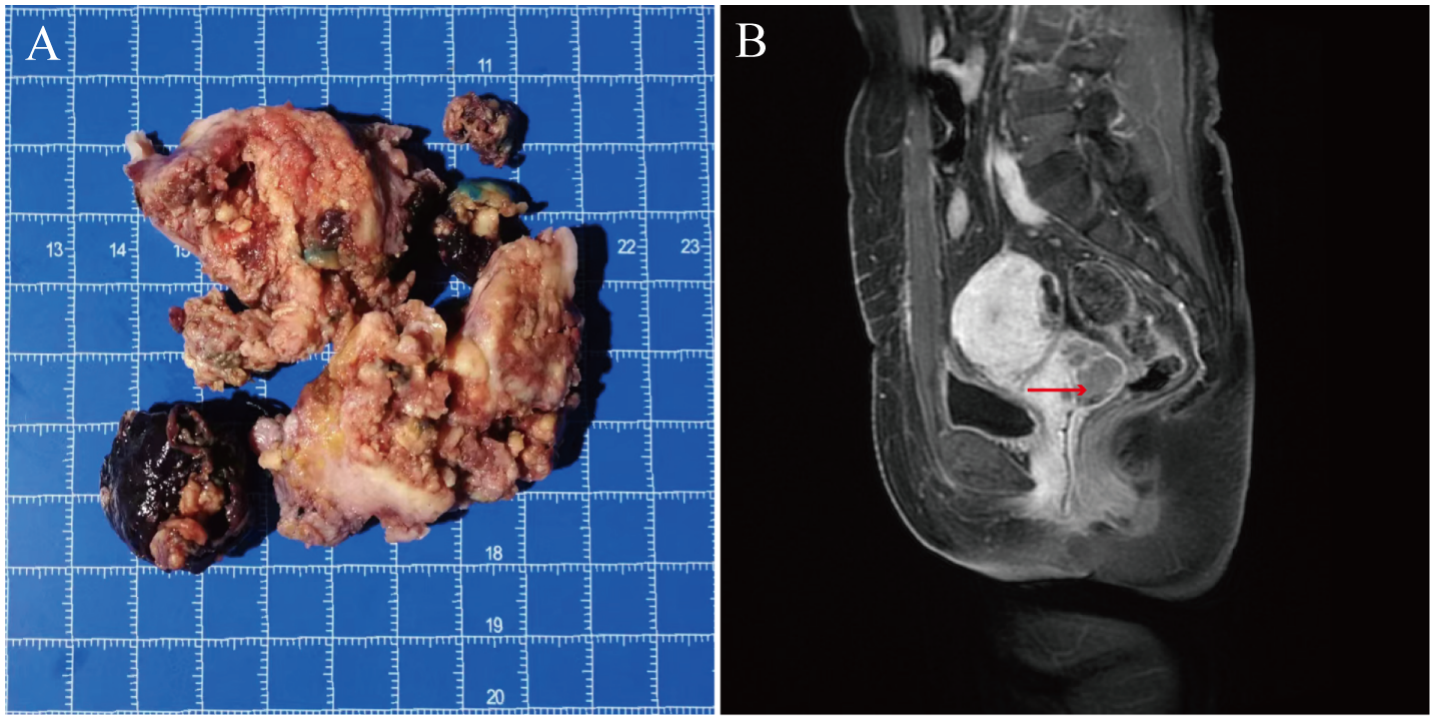
Gross and imaging examination of case 7 with a cervical PEComa. **(A)** The tumor was friable and ill-defined showing yellow to tan fleshy cut surface with necrosis. **(B)** Enhanced MR scan showed the mass located in the cervix measuring a long diameter of about 3.8 cm.

Grossly, all tumors were nodular, round or oval, unencapsulated, well-demarcated or demonstrated varying degrees of infiltrative growth. They were typically single, but multiple nodules were present in individual cases. They ranged widely in size from 3.8 cm to 16.6 cm with a mean of 6.8 cm (median: 5.5 cm). They had tan-pink or yellow-tan cut surface, with some especially the malignancy displaying hemorrhage and necrosis. They were fleshy and soft to firm (**Figure 1**).

**Figure 2:**
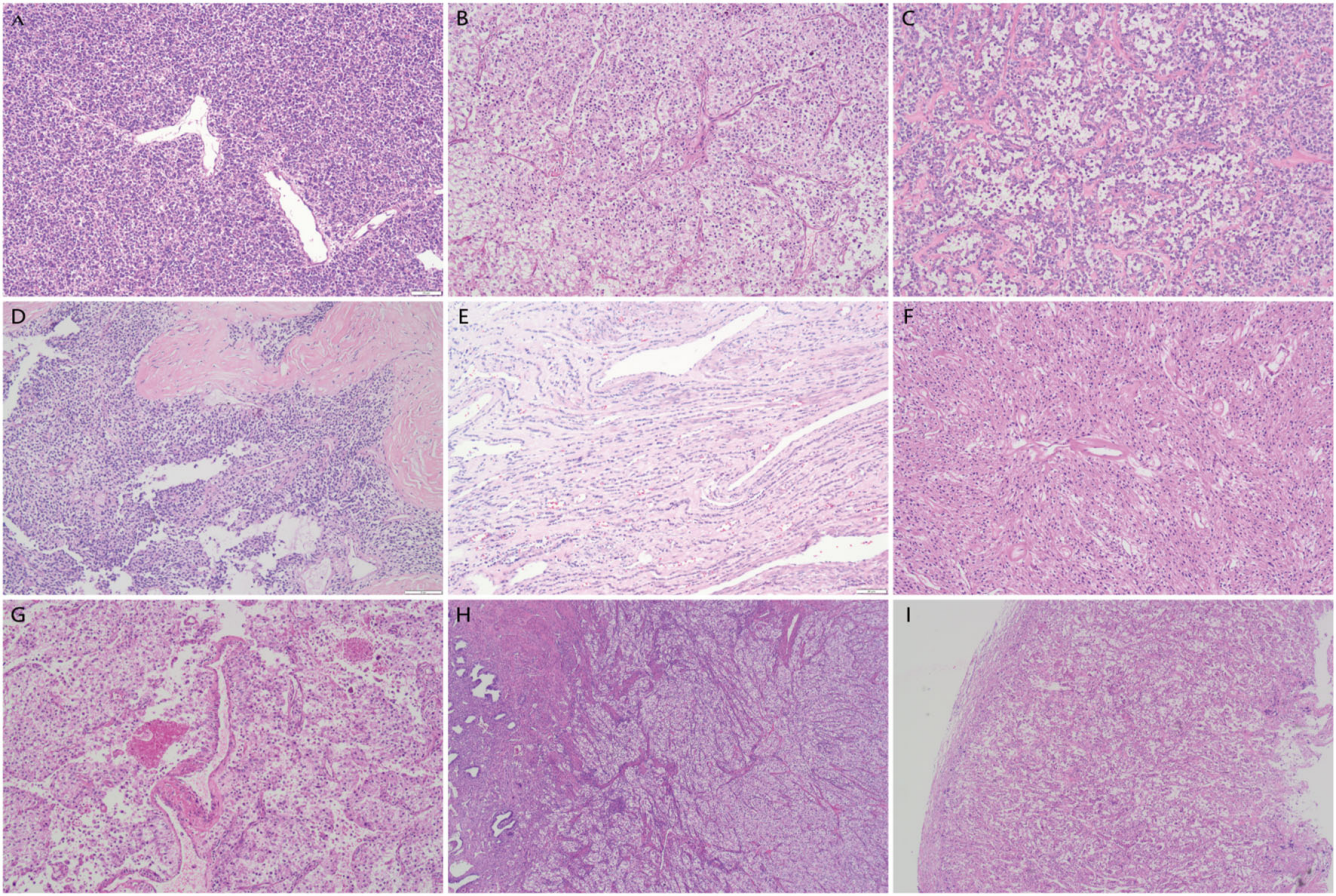
The histology of gynecologic PEComas. **(A)** Perivascular distribution of tumor cells in a solid pattern. **(B)** A perivascular arrangement of tumor cells in nested pattern. **(C)** The tumor cells in a pseudoalveolar pattern. Note the delicate, capillary-like vasculature showing perivascular hyalinization. **(D)** Tumor cells in diffuse growth pattern. Occasionally, patchy or extensive stromal hyalinization may present. **(E)** Tumor cells arranged in cords and emerged in hyalinized stroma. **(F)** A uterine PEComa mainly composed of spindle cells showing radial distribution around the vessels with the central hyalinized arteriole. **(G)** Neoplastic cells arranged around a vascular space and replacing part of the vessel wall. Necrosis was present. **(H)** A tumor showing typical destructive infiltration of the myometrium and endometrium. **(I)** A PEComa with pushing border.

**Figure 3:**
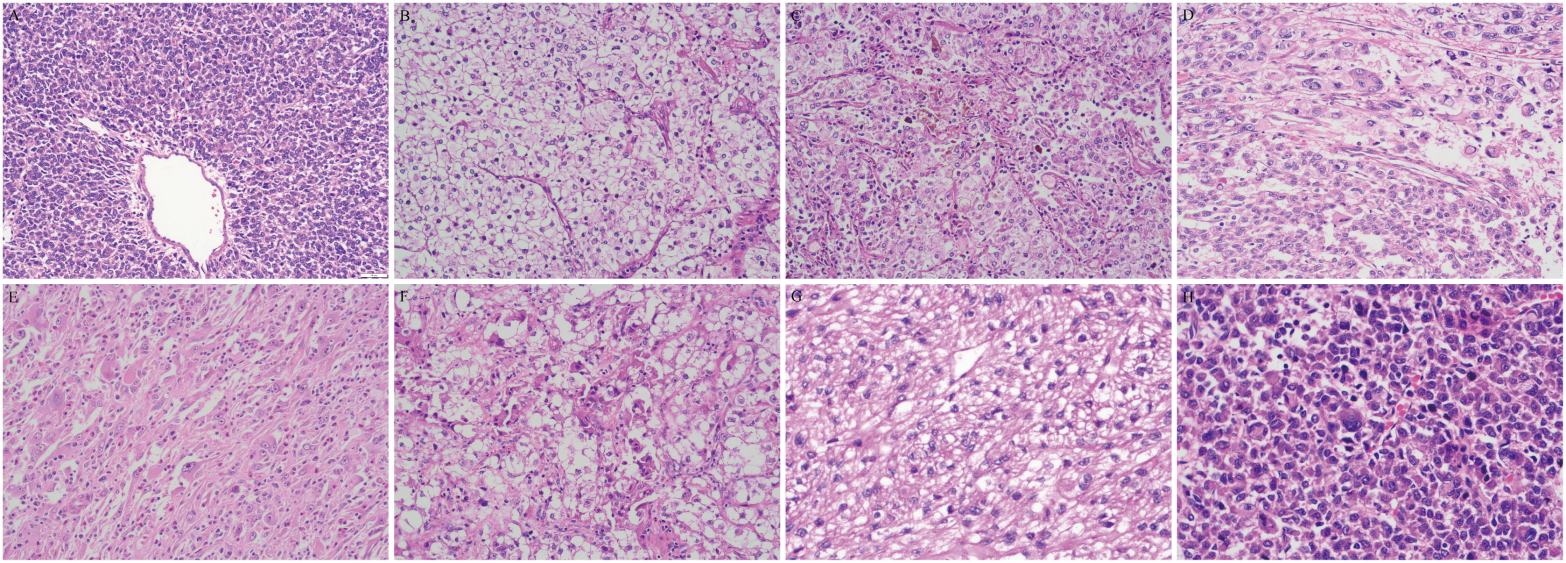
The cytomorphology of gynecologic PEComas. **(A)** Case 4 composed of epithelioid cells with granular eosinophilic cytoplasm. **(B)** Case 2 composed of epithelioid cells with ample clear cytoplasm. **(C)** Intracytoplasmic melanin pigment. **(D)** Intranuclear inclusions and multinucleated giant tumor cells. **(E)** Tumor cells with melanoma-like macronucleoli in an inflammatory stroma. **(F)** Osteoclast-like giant cells and spider cells. **(G)** Tumor cells with mild atypia. **(H)** Tumor cells with significant atypia.

Microscopic examination revealed that most of the tumors (7/9) displayed dyscohesive and epithelioid neoplastic cells arranged in nests, sheets, pseudoalveoli, trabeculae or cords surrounding thin-walled blood vessels, while only two cases were mainly composed of spindled cells in fascicles (**Figures 2A-F**). A perivascular radial growth pattern was prominent in most cases, particularly around larger vessels with the tumor cells replacing the vessel wall (**Figure 2G**). 5 tumors showed infiltration including 4 malignancy presenting diffuse infiltrative growth and 1 UMP showing focal infiltration with 2 tumor thrombi observed (**Figure 2H**). No tongue-like infiltration was observed. The other two UMPs and two malignancies showed expansile growth with circumscribed, pushing borders (**Figure 2I**). The tumor cells displayed well-defined borders, abundant cytoplasm (clear, foamy, or eosinophilic), and large vesicular nuclei (**Figures 3A, B**). The ample eosinophilic cytoplasm imparted a rhabdoid appearance to the tumor cells (**Figure 3A**). Intracytoplasmic melanin pigment was observed within the tumor in five cases. Four tumors presented heavy intracellular melanin focally (**Figure 3C**), and three showed subtle pigments in individual cells, which could be overlooked without careful examination, while it was indiscernible in the other 2 cases. Nuclear membranes were thickened with nucleoli ranging from inconspicuous to prominent (**Figures 3A-H**); 6 out of 9 cases exhibited medium to large eosinophilic nucleoli which was also called melanoma-like macronucleoli (**Figures 3D, E**). Intranuclear pseudoinclusions similar to those of melanoma were identified in 2 tumors (22.2%) (**Figure 3D**). The cytological atypia varied (**Figures 3G, H**). All malignant cases exhibited moderate to significant nuclear atypia. Occasionally, benign and UMP tumors possessed a few tumor giant cells; however malignant ones (4/6) had frequent tumor giant cells, including multinucleated forms (**Figures 3D, E**). Spider cells were identified in both the UMPs and malignant PEComas. Scattered osteoclast-like giant cells were found in 7 out of 9 cases (**Figure 3F**). Mitotic counts were generally low, ranging from 0 to 40/50 HPF. Tumor necrosis was universally present in malignant tumors (**Figure 2G**), whereas one UMP contained multifocal necrotic areas as well.

The stroma predominantly contained slit-like or sinusoidal thin-walled vessels, and a subset of tumors (4/9, 44.4%) occupied large thick-walled vessels (particularly at the tumor peripheries) or small arterioles that may be hyalinized. A portion of the PEComas (5/9, 55.6%) displayed varying degrees of stromal hyalinization. In some areas, single or small nests of epithelioid cells appeared to be immersed in narrow collagen bands in a notably hyalinized or fibrotic background (**Figures 2C-E**). Inflammatory cells were scattered in some tumors. It is worth noting that 2 malignant tumors exhibited dense inflammatory infiltrates: one dominated by lymphocytes, and another showing mixed lymphocytes, eosinophils, neutrophils, and plasmacytes (**Figure 3E**), which was initially misdiagnosed as IMT. **Table 2** summarizes the pathological features of these nine tumors.

**Table 2:** Pathological features of 9 gynecologic PEComa.

Case#	Classification	Tumor border	Cellular morphology	Cytoplasm	Melanin pigment	Nuclear atypia	Macroucleoli	intranuclear pseudoinclusions	Tumor necrosis	Mitotic figure (/50HPF)
1	M	Infiltrative	Epitheloid	Abundant, E	MOC	Significant	*+*	–	+	2
2	UMP	Infiltrative	Epitheloid	Abundant,C	CSC	Mild	+	–	+	1
3	UMP	Pushing with limited invasion	Epitheloid	abundant, E&C	CSC	Mild	+	–	–	2
4	M	Pushing	Epitheloid	moderate,E	No	Significant	+	+	+	25
5	M	Infiltrative	Epitheloid	Moderate, C	MOC	Significant	–	–	+	40
6	M	Pushing	Mainly spindle	Moderate, C	CSC	Moderate	–	–	+	6
7	M	Infiltrative	Epitheloid	Abundant, E	CSC	Significant	+	+	+	3
8	UMP	Demarcated	Spindle	Abundant, E	No	Mild	–	–	–	<1
9	M	Pushing	Epitheloid	moderate,E&F	MOC	Moderate	+	–	+	1

M, malignant; UMP, uncertain malignant potential; E, esinophilic; C, clear; F, foamy; MOC, minimal in occasional cells; CSC,conspicuous in some cells; No, not identified; HPF, high power field.

### 3.3 Immunohistochemistry results

**Figure 4:**
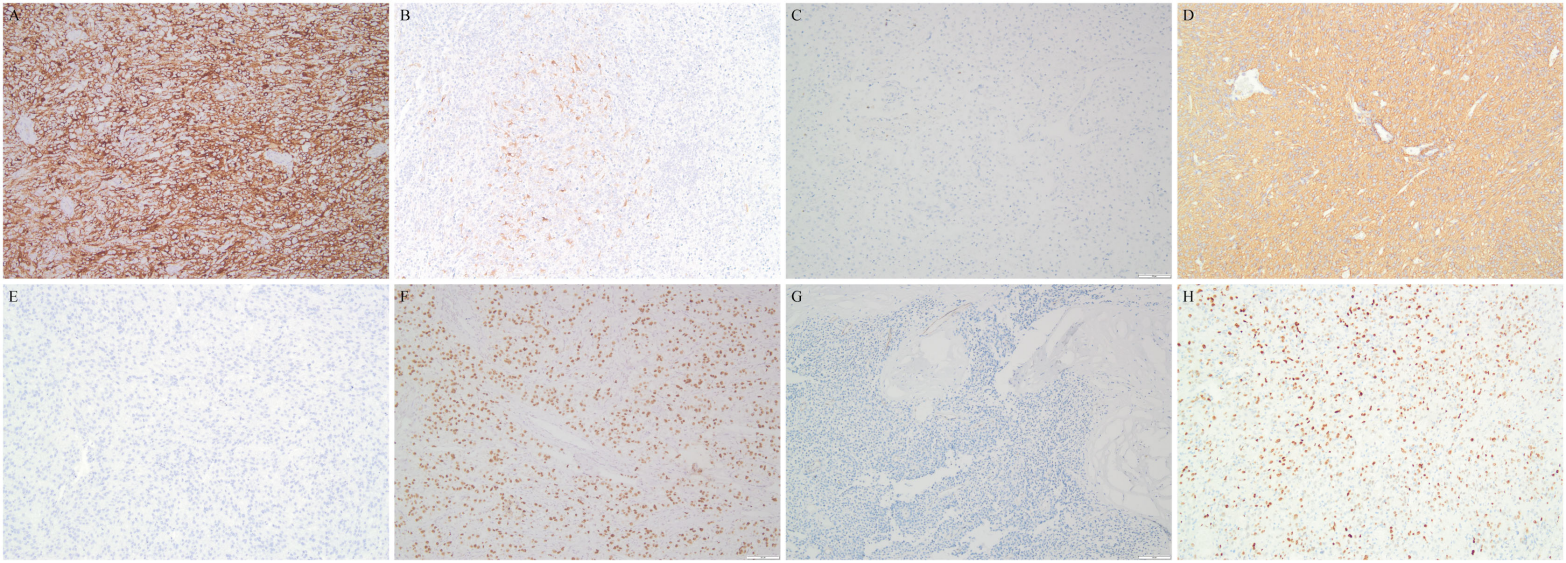
Representative immunostaining of gynecologic PEComas. **(A)** Case 7 demonstrating HMB45 diffuse positivity. **(B)** Case 7 exhibiting focal immunoresponse for MelanA. **(C)** Lack of S-100 in tumor cells. **(D)** Diffuse staining of SMA in tumor cells. **(E)** Totally negative for desmin. **(F)** Typically diffuse positivity of TFE3. **(G)** Absence of Vimentin in this tumor. **(H)** PR positivity present in a subset of gynecologic PEComas.

The IHC results are summarized in **Table 3**. All tumors demonstrated HMB45 immunoreactivity, including 8 cases showing diffuse positivity and only one exhibiting focal expression. Only case 4 displayed diffuse staining of MelanA, and another 3 cases demonstrated very focal reactivity. No S100 staining was observed in any of the nine tumors. Most tumors variably expressed myogenic markers. About half exhibited variable expression of SMA and Caldesmon (44% respectively), whereas desmin was predominantly negative. CKpan was uniformly negative, and only a minority of the cases (2/9) exhibited focal EMA expression. ER and PR showed inconsistent staining patterns in variable intensities. The ki67 index ranged from 3% to 40%, with malignant PEComas generally demonstrating higher ki67 (≥10%). TFE3 exhibited in a diffuse and strong nuclear staining in most cases. Only one tumor showed focal positivity. It is interesting that 5 the tumors were totally negative for Vimentin. The representative photomicrographs were shown in **Figure 4**.

**Table 3:** Immunohistochemistry profiles of 9 gynecologic PEComas.

Case#	HMB45	MelanA	S-100	SMA	Caldesmon	Desmin	EMA	ER	PR	Vimentin	TFE3	ki67
1	++, D	–	–	–	–	–	–	–	–	–	++, D	20%
2	+++, D	–	–	–	–	–	–	–	–	–	+++, D	5%
3	+++, D	–	–	–	–	–	–	–	–	+	+++, D	5%
4	+++, D	++, D	–	++	+,D	+, VF	–	–	+	+	++, D	15%
5	++, F	–	–	–			+,F	–	–	–	+, D	30%
6	++, D	–	–	+++	++,D	++	–	+	–	+	++, D	10%
7	+++, D	++, F	–	–	–	–	–	+	+++	+	++, F	20%
8	++, D	+, VF	–	+++	++,D	–	+, VF	–	++	–	++, D	3%
9	+++, D	+,VF	–	+	++, F	–	–	++	–	–	++, D	8%

D, diffuse; M, moderate; S, strong; F, focal; VF, very foca.

### 3.4 NGS results

**Figure 5:**
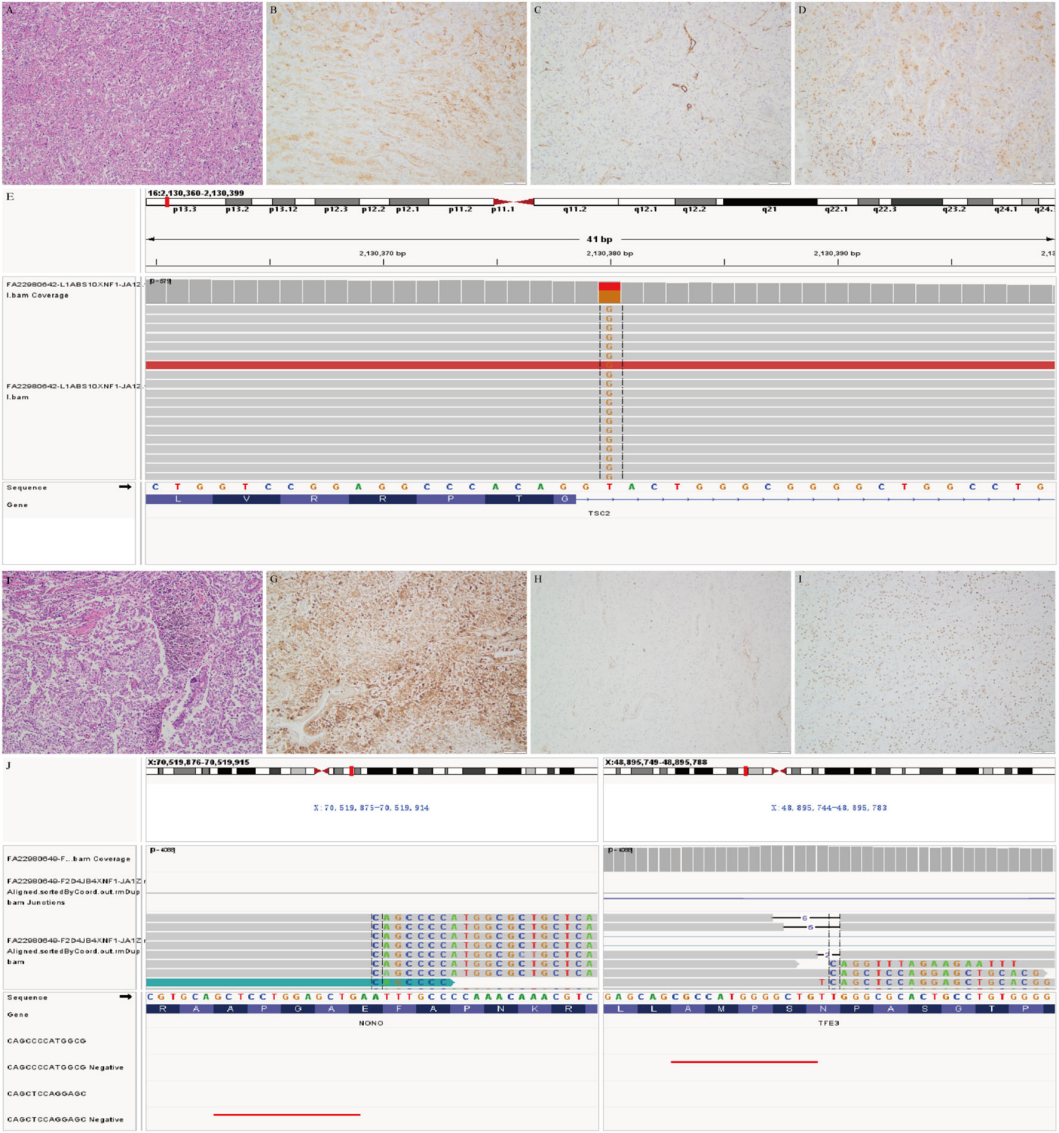
Representative pictures of gynecologic PEComas with genetic alterations. **(A-E)**. This illustrative tumor (case 1) with TSC2 mutation is composed of epithelioid cells with eosinophilic and clear cytoplasm and obvious cellular atypia **(A)** showing HMB45 **(B)** and TFE3 **(C)** immnostaining and absence of SMA **(D)**; Plot of genomic alterations observed in TSC2(c.3610 + 2T>G at intron 30) **(E-J)**. Case 3, this TFE3- translocation associated PEComa, showing heavy melanin pigment in tumor cells with clear cytoplasm and mild nuclear atypia **(F)**, and HMB45 **(G)** and TFE3 **(H)** immnostaining and absence of SMA **(I)**; RNA sequencing revealed NONO (exon 12) fusion with TFE3(exon 4) **(J)**.

Among the 6 tumor cases detected, TSC2 gene alterations were identified in 3 cases. Patient 1 and 8 carried a TSC2 mutation (intron30 c.3610 + 2T>G, exon12 c.1255C>T and intron12:c.1258-1G>T respectively),while patient 4 showed a TSC2 deletion. The other three tumors exhibited TFE3 gene fusions including 1 with TFE3-SFPQ gene fusion, 1 with both NONO-TFE3 and TFE3- IGR (upstream ITGB1BP2) gene fusion, and the other with a novel YAP1-TFE3 gene fusion (**Supplementary Table S1**; **Figure 5**). Among the three TSC2-mutated tumors, two were histologically malignant, whereas two out of three with TFE3 fusions were classified as UMP. There were more genetic changes in the three tumors carrying TSC2 gene alterations than in three tumors carrying TFE3 translocation. Notably that TFE3 gene fusion was only detected by RNA sequencing in 2 tumors. All the tumors undergoing NGS detection were microsatellite-stable (MSS) with low TMB (0–4 mutations/Mb).

### 3.5 Follow-up

Nine patients were followed for 35~62 months (median: 40 months). Among the two non-surviving patients, one died of disease progression, and another died from unexplained thrombocytopenia. Both patients showed TSC2 alterations. Two patients experienced recurrences. One patient with UMP was treated only with local excision due to young-aged and another patient diagnosed as malignant PEComa underwent hysterectomy with bilateral salpingo-oophorectomy followed by short-course radiotherapy without any further therapy. No clinical evidence of tuberous sclerosis complex (TSC) was found.

## 4 Discussion

The histogenesis of PEComa remains unclear because of the lack of an identified normal counterpart. Initially, an association was observed with vascular walls and suggested derivation from “specialized smooth muscle cells” exhibiting myoid and melanocytic morphological features with dual immunophenotypic markers (14, 15). Embryological and *in vitro* studies have proposed an alternative origin of neural crest stem cells capable of differentiating into myoid and melanocytic lineages (16–19), which has been challenged by the lack of S-100/SOX10 expression and focal positivity for NSE and synaptophysin in a small number of tumors. Recently it was proposed to belong to the spectrum of ESMT because both tumors show clinicomorphologic and immunophenotypic overlap, and HMB45 positivity has been recognized in some uterine smooth muscle and normal myometrium (although generally focal and weak) (20–23).

PEComa demonstrates a female predominance, with the uterine corpus being the most frequent site in the female genital tracts. However cases have also been described in the cervix, vagina, ovary, broad ligament, round ligament, and vulva (24). In this cohort of PEComas, 5 originated from the uterine corpus, 3 from the cervix and 1 from vagina respectively. The patients ranged from 6 to 79 years of age, with most in their 6th to 7th decades (25). The 9 patients in this study aged 27–79 years with a mean age of 51 years. Clinically, patients with PEComas are often asymptomatic or are incidentally detected by imaging. Common manifestations include abnormal uterine bleeding, a uterine mass, and abdominopelvic pain (26). A subset of PEComas (~10%) is associated TSC (8, 27, 28). It has been reported that the majority of PEComatosis occurred in patients with TSC and TSC-associated loss of function of TSC1/TSC2 could lead to sclerosing PEComa (8, 29, 30).However, TSC was not presented in this series. It is noteworthy that one old patient was found to have thrombocytopenia and died of its complications. Rothenberger et al. reported one old female diagnosed with uterine PEComa who presented with severe thrombocytopenia and disseminated intravascular coagulation (DIC) (31). The patient underwent hysterectomy after recovery from her DIC (31).

These neoplasms display a broad spectrum of sizes ranging from 0.2 to 25 cm in diameter (average 5–8 cm), and typically present as solitary lesions. Generally malignant PEComa exhibits a larger mass and lacks a capsule with either a well-defined border or an infiltrative growth pattern. Radiological examination usually reveals a mass or indicates as a leiomyoma. Herein, neither the radiologic appearance nor gross features of PEComas are sufficiently distinctive to allow preoperative diagnosis.

Based on previous reports and our experience, gynecologic PEComas demonstrate histology comparable to that of their counterparts in other anatomical sites. The tumors are composed of PECs with abundant clear to flocculant eosinophilic cytoplasm and well-defined cell borders arranged in sheets and nests, less frequently in pseudoglandular pattern, cords, or short fascicles (8, 23–25, 28, 29, 32). Although epithelioid cells are characteristic, a given lesion may be dominated by spindled cells. The neoplastic cells typically exhibit a perivascular distribution. At least focal radial growth around blood vessels may be observed. In most cases, the vasculature predominantly consists of thin-walled capillaries or slit-like sinusoids vessels, however thick-walled large vessels may be scattered, especially at the peripheral regions. Notably, some cases demonstrate a transition between neoplastic cells and vascular walls, with replacement of vascular structures extending to the subendothelial layers, which prompted the diagnosis of PEComa. Variable amounts of delicate, banded, or patchy collagen are usually present in the stroma. Some tumors display perivascular hyalinization of the arterioles. The features that have been described in other articles were observed in select cases including multinucleated tumor giant cells, spider cell-like giant cells, osteoclast-like giant cells (also called Doton giant cells in some studies, which appear to be degenerative in nature), intranuclear pseudoinclusions and intracytoplasmic pigment (8, 29). The size of nucleoli increases with the tumor malignancy. Melanoma-like macronucleoli are common particularly in malignant tumors. In our cohort, all malignant cases demonstrated tumor necrosis and significant nuclear atypia, suggesting that these features serve as strong indicators of progressive biological behavior. Overall, mitotic activity remains low in PEComas overall, although more mitotic figures characterize malignant lesions. A subset of cases may present with prominent inflammatory infiltrates, necessitating a differential diagnosis from IMT or sarcomas.

PEComas demonstrate an immunophenotype characterized by co-expression of melanocytic and smooth muscle markers. In current study, all tumors exhibited HMB-45 immunoreactivity, predominantly with diffuse cytoplasmic staining patterns. Melan-A staining was only observed in approximately 40% of tumors with a very focal or scattered pattern, whereas it has been reported in the majority of PEComas with 74~88% positivity (25, 29). The results confirm that HMB-45 serves as a better marker than Melan-A in PEComa identification, and diffuse HMB-45 staining coupled with the suggested morphology strongly supports the diagnosis. Notably, other melanocytic markers such as S-100 and SOX10 were generally negative. Myoid markers SMA and h-Caldesmon demonstrated variable expression with consistent positivity, whereas the positivity of desmin was quite low (33%) in this study, which is inconsistent with other’s reports of 76~100% (8, 29). This distinct immunoprofile may facilitate the differentiation of smooth muscle neoplasms. In addition, it was found Vimentin was only detected in 4 tumors, suggesting PEComa does not always express this marker. Previous immunohistochemical studies on PEComa seldom mentioned the expression of Vimentin. Folpe et al. found Vimentin negative expression was present in 2 out of 14 PEComas (5). Fadare (23) reported a low Vimentin-positive rate (56%,10/18) in a literature review, which was consistent with Bourgmayer’s conclusion (36/65) (33). The loss of Vimentin could reflect a shift towards a more epithelial-like phenotype in these tumors, and potentially aids distinction from other mesenchymal neoplasms. Hormonal receptors (ER and PR) observed in selected cases of various intensities may reflect site-specific differentiation. Low ki-67 indices (≤5%) are usually observed in UMP while higher indices (>5%) are observed in malignancy. However, the prognostic significance of ki-67 requires validation in larger multicenter studies. Vang and Kempson have described uterine PEComas as two morphological subtypes (20). Group A mimics the growth pattern of low grade ESS with tongue-like permeation of the uterine wall and is composed of cells with abundant eosinophilic or clear cytoplasm. Group B is composed of epithelioid cells with less abundant clear cytoplasm. Group A tumors exhibit significant HMB-45 and Melan-A expression with only focal positivity of smooth muscle markers, while Group B shows less HMB-45 expression, but more extensive muscle marker positivity. However, in our experience, this is not always the case. A subset of PEComas has overlapping morphology and immunostaining profiles. Uterine PEComa, particularly group B, overlaps the clinicomorphology and immunophenotype with ESMT. Some authors have proposed that uterine PEComa is not a distinct entity, but rather was an ESMT with melanocytic marker expression. Katsakhyan and his colleagues have described a series of uterine leiomyosarcomas associated with PEComa showing identical genetic alterations, therefore, they advocated that modified smooth muscle cells represented the origin of a subset of PEComas (34). Based on this scenario, we agree that they may represent a spectrum where smooth muscle tumor always display myomatous differentiation and occasionally display melanocytic differentiation (23), whereas PEComas always display melanocytic differentiation and inconstantly displays some myomatous differentiation. Nevertheless, application of melanocytic markers, to include at least HMB-45, should be considered in all cases of uterine mesenchymal tumors with prominent epithelioid morphology.

PEComas currently comprise two mutually exclusive molecular subgroups, TSC1 or TSC2 alterations and TFE3 fusions. In the current study, six cases were analyzed by DNA&RNA hybrid capture-based comprehensive genomic profiling. We identified TSC2 mutations and TFE3 fusions in all detected tumors, which were mutually exclusive and consistent with previously studies. Further, PEComas harboring TSC2 mutations seem to harboring more additional gene mutations than those with TFE3 fusions. Patients carrying more concurrent gene mutations, such as TP53, ATRX, P63, MLH3, and ARID1A, usually have adverse outcomes. The nonrecurrent mutations, such as TP53, RB1, CDKN2A, ATRX, NF1, FH, RUNX1, MLH3, NOTCH2 and SMARCB1, have been reported in the PEComa family from different sites (10, 35–38). The gene mutations we discovered in this study including BRD4, SRSF2, TGFBR2 and CHD2, have not been previously reported before. Agaram et al. identified coexisting TP53 mutations were identified in 63% of the TSC2-mutated PEComas (30).

Agaram et al. found TSC2 mutations in 62% of the cases tested and in 80% of the TFE3 fusion-negative cases (30). A separate study investigating the genomic landscape of 31 malignant PEComas by integrated DNA and RNA profiling identified TSC2 mutations in 32% of the cases tested and TSC1 mutations in 10%, with 2 additional cases harboring FLCN mutations (36). Inactivating mutations in the TSC1/2 gene lead to mTOR pathway activation, driving cell proliferation and myogenic differentiation, thereby establishing mTOR as a potential therapeutic target for progressive PEComa. An increasing number of studies have shown mTOR inhibitors therapy presents promising results in advanced cases (35, 39–41). Furthermore, the tumors with TSC2 alterations frequently harbored concurrent mutations such as TP53, ATRX, P63, and ARID1A, which may contribute to their adverse outcomes.

The other three PEComas revealed TFE3 rearrangements with fusion partners SFPQ, NONO and YAP1 respectively. RNA sequencing revealed a novel fusion YAP1-TFE3 which was reported in inflammatory spindle cell PEComas of the lung most recently (1 case by Hsu, 2 by Kojima and 1 by MacDonald respectively in 2025) (42–44). YAP1-TFE3 was detected in case 7, which showed numerous lymphocytes, plasmocytes, and scattered eosinophils, lacked any myoid staining and was classified malignant. In this tumor, the TFE3 breakpoint was at exon 7, which is an unusual breakpoint, and the YAP1 breakpoint was at exon 4. Our findings supported the proposal that PEComas with YAP1-TFE3 recruited significant inflammatory cells and may represent a distinct subset of PEComas from different sites. As described in previous studies, TFE3-rearranged PEComas exhibit a distinct morphology, predominantly composed of epithelioid cells with clear cytoplasm, low-grade nuclear atypia and loss of smooth muscle markers. TFE3, a member of the MiT family of basic helix-loop-helix leucine zipper transcription factors, interacts with MITF, which regulates melanocytes and osteoclasts differentiation (6, 45). This could explain the findings that all tumors with TFE3 rearrangement exhibited prominent cytoplasmic pigmentation. Notably, all TFE3-rearranged tumors occurred in young patients (27, 36 and 47 years old, respectively) who were much younger than those with TSC2 alterations (56,79,73 years old, respectively). Two TFE3 fusion tumors were classified as UMP and there was no sign of recurrence or metastasis on the other patient with TFE3 fusion. Although it has been suggested most PEComas with TFE3-translocation exhibited aggressive behavior (8, 24), frequent recurrence, and poor prognosis (10, 11), our findings indicate that TFE3-translocated PEComas may have a more favorable prognosis. However, due to the limited sample size, statistical testing was not performed to assess the association between the two genotypes (TSC2 vs. TFE3) and phenotypes (malignant vs. UMP). TFE3-rearranged PEComas present elevated transcriptional activity of TFE3 and subsequent induction of the c-Met, AKT and mTOR pathway, and might be nonresponsive to targeted mTOR therapy. However, MET inhibitors (e.g., crizotinib) have been explored for the treatment of TFE3-rearranged PEComas (11, 46–48). In addition, if hormone receptors are positive, aromatase inhibitors can be integrated into the treatment plan for patients in the advanced stage.

Moreover, tumors lacking TFE3 translocation may show diffuse TFE3 immunoreactivity as well, highlighting the limited concordance between TFE3 immunostaining and genetic changes. Therefore, even a diffuse strong staining of TFE3 cannot indicate TFE3 fusion. As thus, TFE3 immunohistochemistry cannot be used as a surrogate for FISH or other molecular detection. In this study, one TFE3 rearrangement was detected by DNA sequencing, whereas the other two were identified via RNA sequencing. This discrepancy was due to TFE3 alterations, which typically involve gene fusions rather than mutations. It is generally acknowledged that DNA-based sequencing has limitations in gene fusion detection, while RNA sequencing can detect cryptic translocations. Although TFE3 fusions have been reported in a small number of PEComas (estimated at up to 23%) (36, 49), our data suggest they may be more common than suggested. Due to the small number of cases, we didn’t identify RAD51B gene rearrangement and other fusions. Given the predictive value of TFE3, TSC2, and RAD51B for clinical behavior and tailored therapy, molecular profiling using both DNA and RNA sequencing is recommended for PEComas to guide clinical management.

PEComa represents a category of neoplasms with unpredictable biological behavior, as histologically indolent-appearing cases may demonstrate a clinically aggressive course. Consequently, definitive diagnostic criteria for malignancy remain unestablished, and no validated risk stratification algorithm has been universally accepted. Although several studies have reported a high rate of local control (20, 28, 50, 51), 44% exhibited malignant behavior with resultant mortality or distant metastasis in Fadare’s analysis of 41 uterine PEComas (23), and the majority of the tumors showed malignant or malignant potential in a 16-case series (29). Folpe’s seminal risk stratification system identified 6 histopathological predictors of aggressive behavior including tumor diameter≥5 cm, infiltrative growth, significant nuclear atypia, mitotic activity >1/50 HPF, presence of tumor necrosis, and lymphovascular invasion. Based on these parameters, PEComas are tripartitely classified into the benign, UMP, and malignant categories (28). The malignant designation requires≥2 worrisome features, and UMP is recognized for tumors demonstrating either marked nuclear pleomorphism/multinucleated giant cells or isolated size >5 cm, while tumors having none of these morphologic criteria are qualified as benign. Schoolmeester et al. subsequently examined at prognostic parameters for gynecological PEComas and increased the threshold to four morphologic criteria for diagnosing malignant PEComa (29). Then, Conlon et al. revised the Folpe’s classification system in 2015 by defining malignant PEComa if necrosis or two or more high-risk features were present (52). In 2018, Bennett et al. applied both algorithms to their study of 32 uterine PEComas with documented aggressive clinical behavior in histologically “benign” PEComas (8). They proposed elimination of the “benign” category from the UMP classification schema, and reduction of required diagnostic criteria for malignancy to three features. These amendments have been incorporated into the 2020 WHO classification (11, 53). The application of Folpe’s algorithm in our series showed the majority of cases demonstrated either overtly malignant characteristics or UMP, with only case 8 classified as benign. Case 3, it should be noted, exhibited vascular invasion with amiable histological features, thereby reinforcing the emerging consensus that PEComas should be universally regarded as possessing either confirmed malignant potential or indeterminate biological behavior.

## 5 Conclusions

In summary, we described the salient clinicopathological and genetic features of a cohort of gynecologic PEComas. This rare tumor is composed of histologically and immunohistochemically distinctive PECs. PEComas demonstrated constant melanocytic differentiation, however, varying degrees of smooth muscle responsiveness were observed. HMB45 was confirmed as the most sensitive diagnostic marker. Diagnosis should be confirmed by performing immunohistochemical markers including HMB45, Melan-A, SMA, desmin, S-100, CK, and ki-67. Two mutually exclusive genetic abnormalities, TSC2/1-inactivation and TFE3 rearrangements were identified in all tumors detected by NGS. Additional TFE3 rearrangements may be explored by the integrated DNA/RNA sequencing. We believe that most PEComas have malignant potential and malignancy. Pathological reports should document high-risk features including tumor size, cytological atypia, infiltration, vascular invasion, mitotic activity, and necrosis. Given the limited sample size of this study, the observed phenomena may be subject to some degree of contingency. When molecular analysis is necessary, we advocate a comprehensive genetic profiling at both the DNA and RNA levels to facilitate prognostic evaluation and guide targeted therapy.

## Data Availability

The datasets presented in this study can be found in online repositories. This data can be found in the SRA (https://www.ncbi.nlm.nih.gov/sra/PRJNA1398009) with the accession number SUB15910901.
