# Supplementary material for: Clinicopathological and molecular features of gynecologic perivascular epithelioid cell tumors: a single-center study
Source: Front Oncol. 2026 Feb 18;16:1769702. doi: 10.3389/fonc.2026.1769702 (PMC12956540; doi:10.3389/fonc.2026.1769702)
Supplement: Supplementary file 1 [file DataSheet1.docx]

**Supplementary Materials and methods**

**DNA-based NGS**

Targeted-capture [NGS](https://www.sciencedirect.com/topics/biochemistry-genetics-and-molecular-biology/next-generation-sequencing) was performed with DNA using a 481-gene panel for soft tissue (SARCOPACT™, Geneseeq Technology Inc., Nanjing) to detect single nucleotide variants (SNVs), small insertions and deletions (indels), copy number variations (CNVs), and selected gene fusions, tumor mutation burden (TMB), micro satellite instability (MSI). DNA was extracted from the tissue samples using GeneRead DNA FFPE Kit, then qualified with QIAseq DNA QuantiMIZE Assay Kit (QIAGEN, Valencia, CA, USA). The DNA samples were pooled together for enrichment using 481 gene probes followed by sequenced on the Illumina NovaSeq4000 platform with 2×150 bp double-ended sequences. Finally, comprehensive genetic mutation information was obtained using GATK software.

**RNA-based NGS**

Targeted RNA sequencing was performed using a 149-gene panel for soft tissue (Sarcorna™, Geneseeq Technology Inc., Nanjing) to detect significant gene fusions. The total RNA was extracted using the RNeasy FFPE Kit. The sequencing library was constructed using the KAPA Stranded RNA-Seq Library Preparation Kit. Then, the library underwent targeted hybridization capture of transcripts from 149 genes and was sequenced on the Illumina HiSeq NGS platform (Illumina, San Diego, CA, USA). Base calling was performed on bcl2fastq v2.16.0.10 (Illumina) to generate sequence reads in FASTQ format. The qualified reads were then aligned to the human genome Hg19 (hs37d5) using STAR to identify mutations in individual exons and introns. The average coverage of mapped reads at the feature base positions was calculated. Gene fusions were identified and visualized using the Integrative Genomics Viewer.
