# Supplementary material for: Clinicopathological and molecular features of gynecologic perivascular epithelioid cell tumors: a single-center study
Source: Front Oncol. 2026 Feb 18;16:1769702. doi: 10.3389/fonc.2026.1769702 (PMC12956540; doi:10.3389/fonc.2026.1769702)
Supplement: Supplementary file 2 [file Table1.docx]

**TABLE S1.** **Genetic alterations in 6 gynecologic PEComas**

| **Case#** | **Inactive****TSC2 alteration** | **TFE3** **fusion** | **Other gene mutation** | **MSS** | **TMB** |
| --- | --- | --- | --- | --- | --- |
| 1 | Intron 30 :c.3610+2T>G |  | ATRX, BRD4,TP53 | Yes | 4 |
| 2 |  | TFE3-SFPQ | FH | Yes | 1 |
| 3 |  | NONO-TFE3 & TFE3-IGR (upstream ITGB1BP2) |  | Yes | 3 |
| 4 | TSC2 deletion |  | ARID1A, RUNX1, SRSF2, TGFBR2 | Yes | 0 |
| 7 |  | YAP1-TFE3 | MLH3, NOTCH2 | Yes | 0.83 |
| 8 | Exon12:c.1255C>T ,intron12:c.1258-1G>T |  | CHD2 | Yes | 3.33 |

**TABLE S2. Molecular subtype and clinicopathological features of in 6 gynecologic PEComas**

| **Case#** | **Genetic alterations** | **Age** | **Classification** | **SMA** | **Caldesmon** | **Desmin** | **Ki67** | **Follow-up status** |
| --- | --- | --- | --- | --- | --- | --- | --- | --- |
| 1 | TSC2 mutation | 56 | M | - | - | - | 20% | DOD |
| 4 | TSC2 deletion | 79 | M | ++ | + | + | 15% | DOD |
| 8 | TSC2 mutation | 73 | UMP | +++ | ++ | - | 3% | Alive, NED |
| 2 | TFE3 fusion | 27 | UMP | - | - | - | 5% | Alive, LR |
| 3 | TFE3 fusion | 36 | UMP | - | - | - | 5％ | Alive, LR |
| 7 | TFE3 fusion | 47 | M | - | - | - | 20% | Alive, NED |

DOD, died of disease; LR, local recurrence; NED, no evidence of disease;
