# Supplementary figures and images for: Clinicopathological and molecular features of gynecologic perivascular epithelioid cell tumors: a single-center study
Source: Front Oncol. 2026 Feb 18;16:1769702. doi: 10.3389/fonc.2026.1769702 (PMC12956540; doi:10.3389/fonc.2026.1769702)

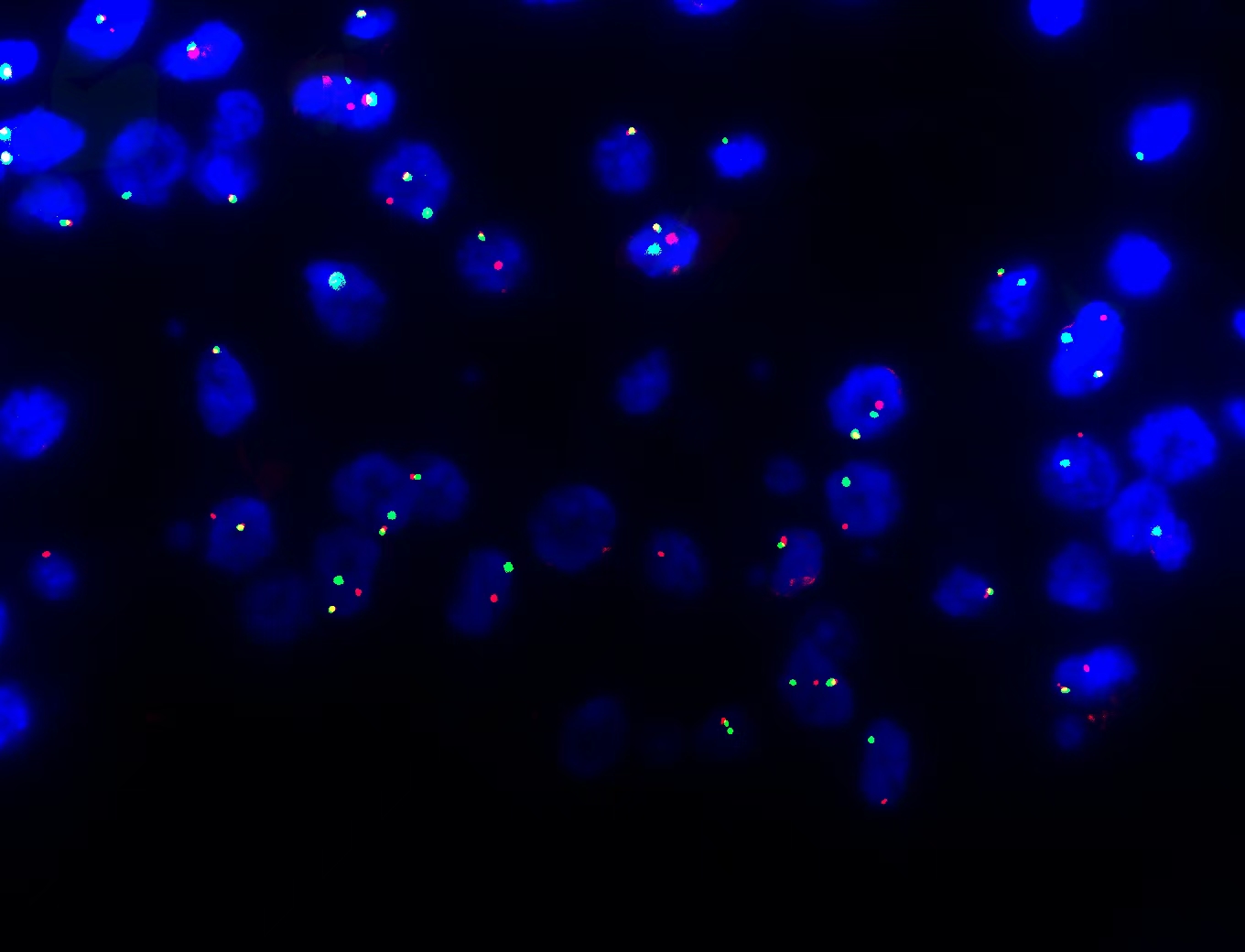

Supplement: Supplementary file 3 [file Image1.jpeg]
